# Relationship of Soil Microbiota to Seed Kernel Metabolism in *Camellia oleifera* Under Mulched

**DOI:** 10.3389/fpls.2022.920604

**Published:** 2022-06-20

**Authors:** Honglian Ye, Yue Wen, Zhigang Chen, Taikui Zhang, Shengxing Li, Menglong Guan, Yunqi Zhang, Shuchai Su

**Affiliations:** ^1^Key Laboratory for Silviculture and Conservation, Ministry of Education, Beijing Forestry University, Beijing, China; ^2^Department of Plant Science, University of California, Davis, Davis, CA, United States; ^3^Research Center for Xinjiang Characteristic Fruit Tree, College of Forestry and Horticulture, Xinjiang Agricultural University, Urumqi, China; ^4^State Key Joint Laboratory of Environmental Simulation and Pollution Control, School of Environment, Tsinghua University, Beijing, China; ^5^Ministry of Education Key Laboratory of Biodiversity Science and Ecological Engineering, School of Life Sciences, Fudan University, Shanghai, China; ^6^Camphor Engineering Technology Research Center for State Forestry Administration, Jiangxi Academy of Forestry, Nanchang, China; ^7^West China Hospital of Sichuan University, Chengdu, China; ^8^Beijing Academy of Forestry and Pomology Sciences, Beijing, China

**Keywords:** association analysis, *Camellia oleifera*, fruit development, metabolites changes, mulching, soil 16S rRNA

## Abstract

An experiment was conducted from 2016 to 2017 to assess the effect of kernel metabolism in development stages after organic mulching compared to control. Organic mulching significantly increased crop yields (higher 128% in 2016, higher 60% in 2017), oil content (the highest oil content was 27.6% higher than that of the control), and improved soil properties (SOC, SAN, AP, and AK). In this study, soil pH, SOC, AN, AP, and AK in 0–30 cm soil depth were measured. Results showed that the effect of mulching on soil pH was not significant at the harvesting stage. The greatest metabolic differences occurred during the period of high oil conversion (S2–S4), primarily involving 11 relevant metabolic pathways. This further verified that *Camellia oleifera* oil yield was improved after mulching. A total of 1,106 OTUs were detected by using 16S rRNA, and Venn diagram showed that there were 106 unique OTUs in control and 103 OTUs in the treatment, respectively. Correlation analysis showed that soil pH and soil temperature were two indicators with the most correlations with soil microbiota. The yield was significantly positively correlated with soil microbial Proteobacteria, Bacteroidetes, and soil nutrition indexes. Organic mulching improved the physicochemical properties of soils, caused differences in the relative abundance of dominant bacteria in soil bacteria, and improved the soil microbiological environment to promote plant growth, indicating that organic mulching is an effective measure to alleviate seasonal drought.

## Introduction

Soil is an essential substrate for terrestrial plants, and it has a diverse microbial community. Some of these microorganisms play key roles in plants, such as assisting plant growth by improving nutritional status or suppressing soil pathogens (Van Der Heijden et al., 2008). Soil biota directly or indirectly affect the plant metabolome by triggering plant responses (e.g., induction of resistance systems; van de Mortel et al., 2012). Climate and weather-related stresses also affect metabolism. For example, certain metabolites are induced in peas during drought stress in their leaves (Charlton et al., 2008). Similarly, changes were observed in the proteome and metabolome of xylem sap in maize plants under drought stress (Alvarez et al., 2008). Soil properties also affect the composition of plant metabolites. Salinity tension, for example, raises the number of nitrile and cyanide-containing compounds (Johnson et al., 2003), and sulfur concentration has a direct impact on mustard oil accumulation (Falk et al., 2007). Nitrogen levels in the soil have a significant impact on plant metabolomes, affecting anything from amino acids to carbohydrates and secondary metabolites (Urbanczyk-Wochniak and Fernie, 2005). Substantial changes in the plant metabolome can also result from other factors, including the pH of the soil, soil texture, moisture, and additional environmental factors (Badri et al., 2013).

Under the ongoing pressure of increasing climate change, it is important to define and explain the relationship between plants and microorganisms in response to abiotic stresses (Classen et al., 2015). Researchers have shown that properties of plant leaves differ depending on the soil environment (Ristok et al., 2019) and can be explained by differences in soil abiotic properties. Important soil properties to consider for plant growth include water use efficiency, nutrient content, and differences in the soil microbiota (Adesemoye et al., 2008; Pinto et al., 2010; Zhou et al., 2022). *C. oleifera* originated in China and is widely cultivated for unsaturated fatty acids in its seed kernels, which are beneficial to humans. However, its production is threatened by seasonal droughts. Mulching is an effective technique in crop production and is primarily used to improve the soil environment so that plants can cope with adverse environmental conditions. Inorganic mulching is commonly used in many agricultural production areas, where drought is an issue, as a cheap and water-saving measure. Although mulching improves soil humidity and prevents soil loss, it can alter soil biology and negatively affect soil quality and sustainability (Wang et al., 2009; Ni et al., 2016). Organic mulches from plant residues are better for soil health and are less prone to negatively influence soil quality than inorganic mulches. For example, organic mulches also reduce soil water evaporation, conserve soil moisture, and suppress weed growth. Additionally, they provide minerals, increase soil microcosm biodiversity, and provide fertility to the soil and plants (Forge et al., 2003; Yang et al., 2003; Kołota and Adamczewska-Sowińska, 2013).

Soil composition needs to be considered when optimizing agricultural practices to promote the growth of the edible oil producing tree *C. oleifera*. The metabolic characteristics of the fruit development stages in the main growing areas under prolonged seasonal drought conditions and the links between the soil microbiome and the field *C. oleifera* fruit seed kernel metabolome after organic ecological mat mulch treatment is not clear. We aimed to determine how mulching influences the metabolic characteristics of the fruit at the fruit development stages for *C. oleifera* trees by organic mulching and the relationship between the yield with soil microbes. The treatment is based on our previous research results (Ye et al., 2021b). The plant metabolome is chemically diverse, and it is unlikely that the metabolism of the plant will change in one or more specific compounds and groups. Therefore, the chemical response of *C. oleifera* to mulching was studied with a nontargeted metabolomics approach. This study also investigated the influence of ecological mulching on the soil microbiota using 16S rRNA sequencing. In addition, the effects of low-cost biodegradable mulch materials on soil nutrition, crop growth, and yield were investigated in this study.

## Materials and Methods

### Field Experiment

The experiment was conducted in June 2016 at Jiangshan Company site in Changning, Hunan Province, located at 112° 40′ 00″ E26° 42′ 09″ N. The soil of the trial site was an acidic red soil (Gong, 1999). The area has a typical subtropical monsoon climate with a mean annual precipitation of 1,400 mm and a summer rain probability of less than 14%. Rainfall was mainly in January–June, and December, and there was severe seasonal drought that lasted for 5 months. Summer temperatures reached up to 44°C in August, and the lowest temperature was −2°C in January. The climate conditions of the test site in 2016–2017 refer to previous research (Ye et al., 2021a). Fruit canopy yield per unit area (kg/m^2^) = weight of the whole tree fruit (kg)/canopy area (m^2^) at harvesting.

This experiment used 6-year-old *C. oleifera* trees (drought resistant) with the following treatments: (1) CK: no mulching (*n* = 30 trees). (2) FG (Mulching): Mulching (*n* = 30 trees). Mulching time from June 2016 to 2017 until sampling. The mat material contained various plant materials (was named ecological mat), with a thickness of 3–4 cm (Chen et al., 2016; Ye et al., 2021a). Each plot of trees was 80 m^2^ under unified management. The experiment was undertaken under rainfed and without irrigation. The species was xianglin210, with a distance of 2.5 m between rows and 1 m between plants.

### Soil Sampling and Soil Condition

Interroot soil collection was carried out during the fruit ripening period (October). The root system is concentrated in the soil layer of 0–40 cm and accounts for 70–80% of the total root system (Yuan et al., 2009). Considering the root distribution of *C. oleifera* and the difficulty of soil collection, soil from around the roots was collected within 20 cm * 20 cm of the tree trunk. All samples of each block were taken from the topsoil (0–30 cm) and fully mixed to form a composite sample. An aluminum spoon was used to gently collect the periroot soil, which was passed through a 2-mm sieve to homogenize the soil sample to discard above ground material (plant residues and stones). Triplicate soil samples from each treatment were stored at −80°C to extract total soil DNA for high-throughput sequencing (Liang et al., 2015; Wang et al., 2015). The rest of each sample was gently air-dried and used for physicochemical measurements. Physicochemical parameters, e.g., pH, organic carbon, available N, P, and K, were measured for each composite sample. The pH was determined to the methods of previous studies (Sun et al., 2015; Qi et al., 2017). The available nitrogen (AN, alkalized nitrogen method), available phosphorus (AP), and available potassium were determined with Lu’s described procedures (AK, extraction of 1.0 M ammonium acetate; Lu, 2000). The method for the determination of organic carbon was used by Nelson and Sommers (1982). Soil temperature was measured 5 cm below the surface by a right-angle thermometer (YF-303, Yunfei, China), and the soil water potential was measured by a tensiometer (0–85 kPa). For this study, we only need soil water potential during harvest. According to Xu’s patent instructions (Xu, 2011), the tensiometer (clay head) was correctly installed at 30 cm from the soil surface. Because the climate of the test site during the harvest (continuously cloudy) did not change the fluctuation range, water potential was collected every 2 days (at 9 a.m., totally of three times) during the same fruit harvest period.

### Fruit Sampling

The fruit was collected monthly from July to October (S1–S4) 2017. Collect once a month on the 20th. Twelve fruits were collected from each tree. One small, medium, and large fruit were collected from the east, south, west, and north side of each tree. Three fruits were randomly selected from the 12 and the seed kernels were sliced and stored in dry ice. All remaining fruits were used for oil content determination. Seed oil was extracted following the method set forth by Ye et al. (2021a). Fruit canopy yield per unit area (kg/m^2^) = weight of fruit of the whole tree (kg)/canopy projection area (m^2^) at harvesting.

### Gas Chromatography–Mass Spectrometry

Seed kernels were ground and 50 ± 1 mg of sample was added to a 2 ml EP tube. Adonitol (0.5 mg/ml stock in dH_2_O) was added as an internal standard to 480 l of extraction solution (methanol/H_2_O (3:1, v/v)). Tubes were then vortexed for 30 s and homogenized with a ball mill for 4 min (45 Hz). Tubes were homogenized in a ball mill for 4 min at 45 Hz. Following, it was sonicated in ice water for 5 min and centrifuged at 12,000 rpm and 4°C for 15 min. Then, 100 μl of supernatant was aspirated and transferred it to a 1.5-mL EP tube, and 20 μl from each independent sample was mixed to obtain a QC sample. The extract was dried without heating in a vacuum concentrator. Ten microliters of methoxyamination hydrochloride (methoxyamine hydrochlorine, dissolved in 20 mg/ml pyridine) was added; after mixing and incubation for 30 min at 80°C. Then, 100 μl of BSTFA (1% TMCS, v/v) was added and the mixture was incubated at 70°C for 1.5 h. 5 μL of saturated fatty acid methyl ester (dissolved in chloroform) was added to the mixed sample (QC sample) after the vials cooling to room temperature.

An Agilent 7890 gas chromatography-time-of-flight mass spectrometer was equipped with an Agilent DB-5MS capillary column (5% diphenyl, 95% dimethylpolysiloxane; 30 m × 250 μm inner diameter, 0.25 μm film thickness; J&W Scientific, Folsom, CA, United States) for GC–MS data acquisition. One μL of sample volume with a splitless injection. The flow rate of inlet purges was 3 ml min^−1^ with helium used as the carrier gas, and the flow rate through the column was 1 ml min^−1^. The initial temperature ramp was held at 50°C for 1 min, raised to 310°C at a rate of 10°C min^−1^, and held for 8 min. The front injection, transfer line, and ion source temperatures were 280°C, 280°C, and 250°C, respectively. The electron energy was −70 eV. After a solvent delay of 6.03 min, mass spectrometry data were collected in full-scan mode at a mass range of 50–500 m/z and an acquisition rate of 12.5 spectra per second (Ye et al., 2021a).

### 16S rRNA

The Power Soil DNA Isolation Kit (MOBIO Laboratories) was used to extract total bacterial DNA from soil samples. The extracted total DNA was evaluated and stored at −80°C for future use. A common primer pair (forward primer, 5′-ACTCCTACGGGAGAGGCGCAGCA-3′; reverse primer, 5′-GGACTACHVGGGTWTCTAAT-3′) was used for PCR amplification of the bacterial 16S rRNA V3–V4 region. The amplification system included 10 μl buffer, 0.2 μl Q5 high-fidelity DNA polymerase, 10 μl high GC enhancer, 1 μl dNTP, each primer 10 μM, and 60 ng genomic DNA, and ddH_2_O was added to a total volume of 50 μl. The reaction conditions were as follows: denaturation at 95°C for 5 min, and then cycle at 95°C, 50°C, and 72°C for 1 min, for a total of 15 cycles. Finally, it was extended at 72°C for 7 min. VAHTSTM DNA Clean Beads purify the product of the first step of PCR. The second round of PCR amplification was carried out in a volume of 40 μl, which contained 10 μl of the PCR purified product of the first step target area, 20 μl 2 × PhμsionHF MM, 1 μl of each primer and 8 μl ddH_2_O. The reaction conditions were as follows: initial denaturation at 98°C for 30 s, then continuous denaturation at 98°C for 10 s, denaturation at 65°C for 30 s, and continuous denaturation at 72°C for 30 s, a total of 10 cycles. Finally, the cells were denatured at 72°C for 5 min. Quant-iTTM dsDNA HS Reagent quantified and pooled all PCR products together. The Illumina 2500 platform (2–2,550 paired ends) was used for high-throughput sequencing analysis of bacterial rRNA genes on pooled samples that could be purified. The original sequence was managed by Trimmomatic software and merged with FLASH. The collected raw sequence data collection is available on NBCI (SRA: PRJNA698393).

### Statistical Analysis

The soil conditions, fruit yield, and metabolite abundance between the treatment and control groups were determined out using Student’s *t*-test. The raw GC metabolic spectrum data was processed using LECO Corporation’s Chroma TOF 4.3X software and the LECO-Fiehn Rtx5 database, including baseline filtering and calibration, peak comparison deconvolution, and peak identification. Mass spectrometry matching and retention index matching were considered in metabolite identification. KNN was used to estimate missing values (featurewise) and to delete features with missing values of more than 50%. Multivariate statistical analysis (PLS-DA) was used for metabolic data analysis, using UCLUST (Edgar, 2010) in QIIME (Ramírez-Guzmán et al., 2004; version 1.8.0) to perform tags with 97% similarity clustering to obtain OUTs. The biological role of different metabolites at each growth stage was determined using pathway analysis. The known metabolite abundances were analyzed using MetaboAnalyst pathway enrichment features, using *Arabidopsis thaliana* as the KEGG reference pathway. The alpha diversity index of the samples was calculated using Mothur (version 1.30) software. Linear discriminant analysis (LDA) and effect size (LSe) analyses were combined with LEfSe software,^1^ to identify significantly different biomarkers between treatments. Correlation analysis based on the phylum-specific abundance of each soil sample was used to determine how various soil microbial communities were associated with soil nutrient indicators and plant traits (oil content, yield, and seed kernel metabolites). The Wukong platform^2^ was used to analyze the relationship between soil microbial community structure, soil condition factors, fruit yield, and metabolites through Spearman correlation, and Cytoscape (version 3.8. 0) visualization was performed.

## Results

### Metabolic Changes During the Development of *Camellia oleifera* Fruit

#### Metabolic Analysis

**Figure 1:**
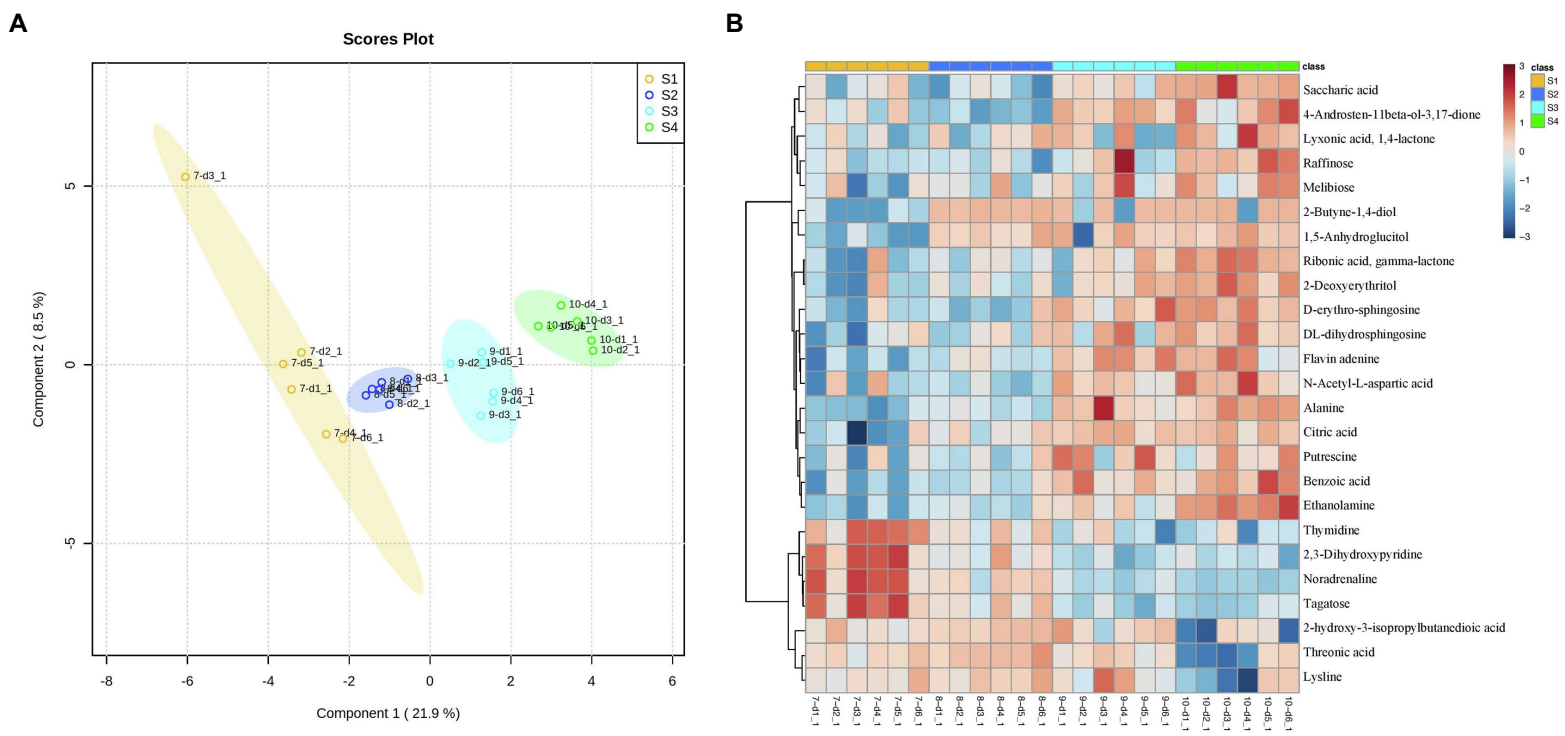
**(A)** PLS-DA score plot for four stages (S1–S4) of *Camellia oleifera* fruit development; **(B)** heatmap of the top 25 metabolites with the highest peaks at four stages of *C. oleifera* fruit development.

A total of 629 unique peaks were detected using GC-TOF-MS, of which 189 were annotated as known metabolites and the rest as unknown metabolites. The known metabolites included amino acids, organic acids, sugars, sugar alcohols, amines, carbohydrates, and phenolics. PLS-DA was conducted at four-time points for *C. oleifera* grown under natural conditions. The first principal component (PC1) in the score plot explained 21.9% of the total variation, and the second principal component (PC2) explained 8.5% of the variation in the entire data set (Figure 1A). This analysis revealed a clear difference in metabolite accumulation at the four-time points (S1, S2, S3, and S4) sampled. Two-by-two comparisons of each stage were also separated from each other (Supplementary Figure 1), indicating differential metabolite profiles between the two stages (S1–S2, S2–S3, S3–S4).

#### Cluster Analysis

The abundance of metabolites reflects the relative content of metabolites. Figure 1B shows the top 25 metabolites in terms of relative content. Based on the direction of change of metabolites during fruit development, these 25 metabolites can be divided into two clusters. The first cluster had 18 metabolites (four sugars, three amino acids, four amines, one sugar alcohol, one carbohydrate, one benzoic acid derivative, one steroid, one organic acid, one nucleotide, and one diol), which were the top 18 metabolites shown in the heatmap and accumulated gradually with fruit development and reached a maximum at the S4 stage. Cluster two, shown on the bottom 7 metabolites of the heatmap, can be subdivided into two subgroups. Subgroup one contained four metabolites (one purine, one alkaloid, one phenol, and one carbohydrate) which had the highest relative content at the S1 stage and then gradually decrease. Subgroup two contained three metabolites (one carbohydrate and two amino acids), with no significant changes in the S1–S2 phase and then progressively decreased between the S3 and S4 phases. The VIP projections (VIP > 1.5) obtained from the three Groups S1–S2, S2–S3, S3–S4 analyzed using PLS-DA were combined with one-dimensional analysis (value of *p* (t TEST) < 0.05, FC > 1.5 or < 0.5). As shown in Table 1, a total of 18 differential metabolites met these criteria from S1–S2, S2–S3, and S3–S4 with 2, 8, and 8 differential metabolites found, respectively.

**Table 1:** S1–S2, S2–S3, S3–S4 comparison of the screened differential metabolites and their VIP values, fold change and *p*-values.

Compare group	Significant different metabolites	VIP scores (component 1)	Fold change	*p*-value
S1-S2	1,5-Anhydroglucitol	3.23	8.85	8.36E-04
	Noradrenaline	2.49	0.16	3.71E-02
S2-S3	4-Androsten-11beta-ol-3,17-dione	2.62	4.06	2.95E-04
	N-Acetyl-L-aspartic acid	1.59	1.99	3.58E-04
	D-erythro-sphingosine	2.03	2.95	2.82E-03
	Flavin adenine	2.07	2.89	7.66E-03
	5-Dihydrocortisol	1.55	2.11	7.71E-03
	Noradrenaline	2.51	0.28	1.50E-02
	Saccharic acid	1.73	2.32	1.67E-02
	Benzoic acid	1.81	2.50	2.54E-02
S3-S4	Myo-inositol	1.71	1.66	1.85E-03
	Pyruvic acid	2.36	2.96	2.92E-03
	Aspartic acid	2.13	0.27	6.55E-03
	D-Glyceric acid	1.71	1.73	7.64E-03
	Glucoheptonic acid	1.84	2.05	9.92E-03
	Oxalacetic acid	1.57	1.60	3.59E-02
	Phosphate	1.86	0.31	4.30E-02
	D-Glucoheptose	1.52	1.50	4.82E-02

To assess significant differences, a two-tailed paired Student’s *t*-test was used (P < 0.05).

**Figure 2:**
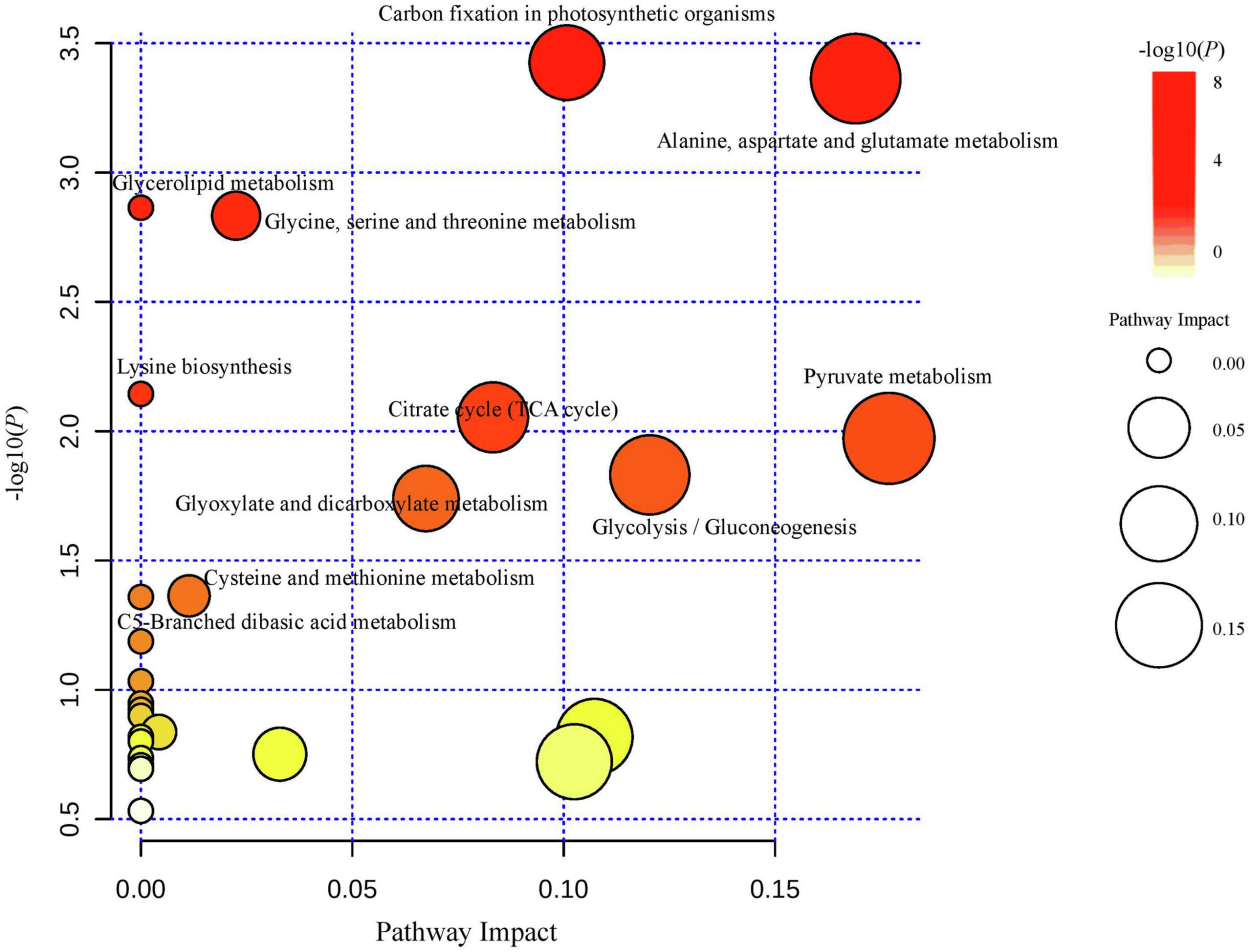
Metabolic pathways involved in the screening of differential metabolites and pathway impact under the comparison of S1–S2, S2–S3, and S3–S4 stages of *C. oleifera* fruit development.

The biological role of different metabolites at each growth stage was determined using pathway analysis. The known metabolite abundances were analyzed using MetaboAnalyst pathway enrichment features, using *A. thaliana* as the KEGG reference pathway. This analysis found 27 differentially regulated pathways at the four stages sampled (Figure 2; Supplementary Table 1). The most significant and highest impact pathways included carbon fixation in photosynthetic organisms; alanine, aspartate, and glutamate metabolism; glycine, serine, and threonine metabolism; citrate cycle (TCA cycle); pyruvate metabolism; glycolysis/gluconeogenesis; glyoxylate and dicarboxylate metabolism; and methionine metabolism; C5-branched dibasic acid metabolism; glycerolipid metabolism; and lysine biosynthesis (Figure 2).

### Analysis of Soil Conditions and Plant Fruit Yield After Mulching

**Figure 3:**
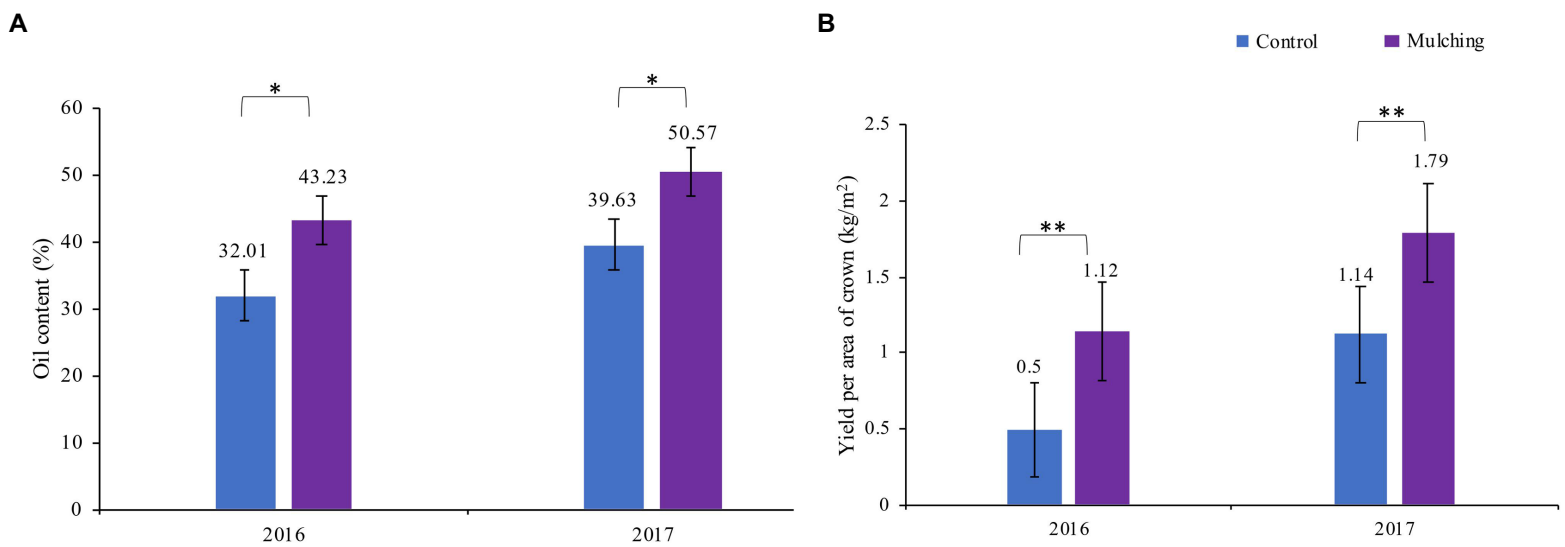
Yield and oil content per unit area of crown area of *C. oleifera* fruit at harvest for two consecutive years from 2016 to 2017. * Represents *p* < 0.05, ** is *p* < 0.01.

The observed oil content of fruit and yield were substantially higher per area of canopy compared to the control in mulched trees (Figure 3) for both years tested (the mulching time from 2016 to 2017). The highest oil content was 27.6% higher than that of the control. The fruit yield was 128% in 2016 and was 59.8% in 2017 higher than that of the control group. The soil properties such as soil water potential, AN, AK, AP, and organic matter were significantly higher in the mulching treatment group after a two-year mulching with the ecological mat than in the control group, but pH and soil temperature were lower in the control group (Table 2).

**Table 2:** Soil properties of *C. oleifera* fields at harvesting after mulching.

	SP (kPa)	ST (°C)	SAN (mg/g)	AP (mg/g)	AK (mg/g)	SOC (mg/g)	pH
Control	−17.33	19.42	141.40	2.03	57.45	15.50	4.73
Mulching	−14.33	19.21	187.87	4.51	75.50	22.43	4.54
Value of *p*	1.24E-03	3.45E-03	4.30E-03	1.20E-03	2.45E-03	3.59E-03	4.87E-03

SP—soil water potential, ST—soil temperature, SAN—available nitrogen in soil, AP—available phosphorus in soil, AK—available potassium in soil, SOC—organic carbon in soil.

### Influence of Mulching on the *Camellia oleifera* Fruit Kernel Metabolome

**Figure 4:**
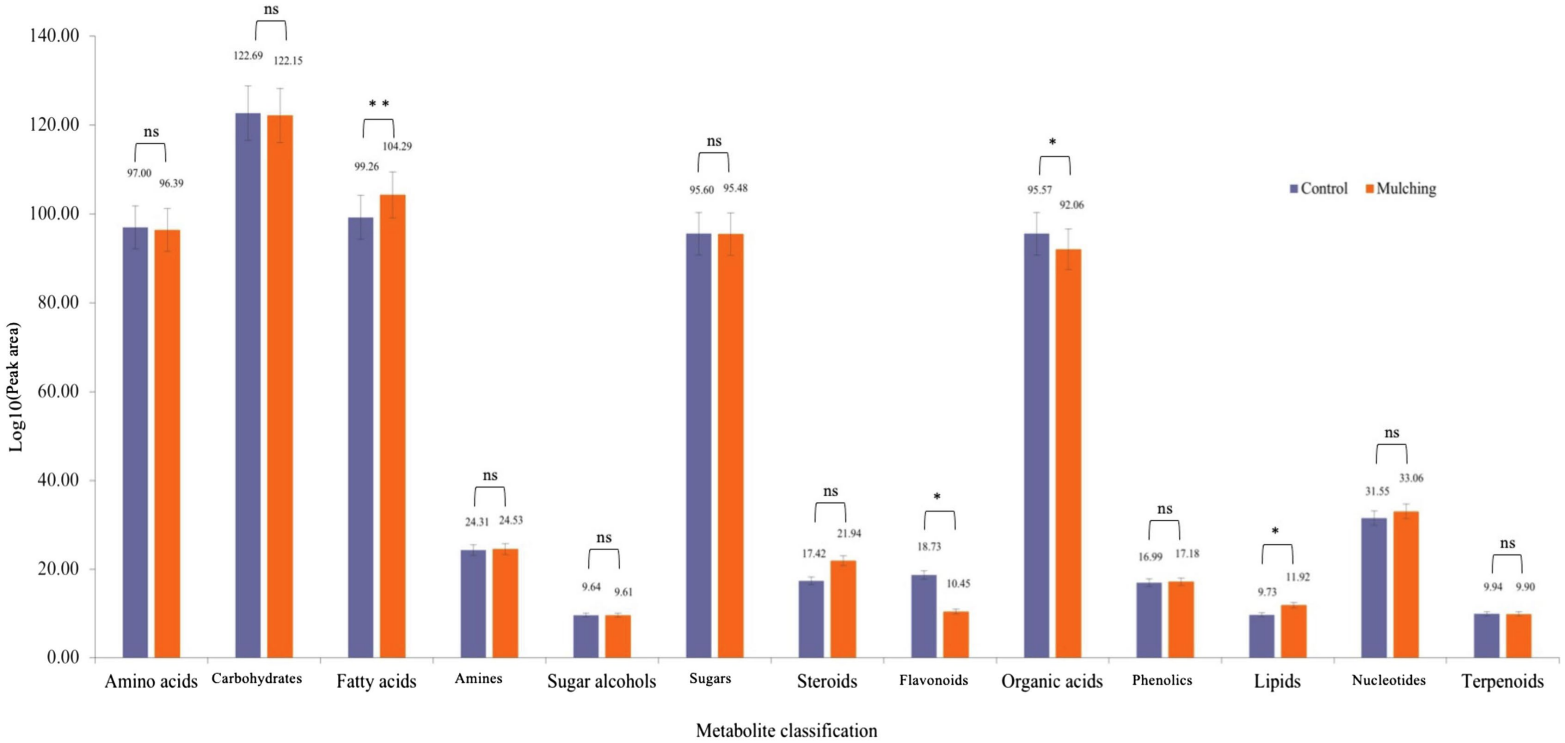
Metabolomics analysis of seed kernels of *C. oleifera* during fruit ripening after mulching. Metabolomics characteristics were detected by GC–MS and classified according to the accumulated peak area of each category. Student’s *t*-tests were used to compare the control and mulched groups. * NS is not significant.

GC–MS analysis of the seed kernels of *C. oleifera* during fruit ripening resulted in annotation of 189 compounds including amino acids, carbohydrates, fatty acids, amines, sugar alcohols, sugars, steroids, flavonoids, organic acids, phenols, lipids, nucleotides, and terpenes. The summed peak areas of compounds in each of the 13 categories were used to determine whether the ecological mat mulching significantly changed any of the metabolite category abundances. The sum peak areas of fatty acids (*p* = 0.0059), flavonoids (*p* = 0.015), organic acids (*p* = 0.043), and lipids (*p* = 0.02) differed significantly from those of the control group after statistical analysis, but not amino acids (*p* = 0.054), carbohydrates (*p* = 0.54), amines (*p* = 0.11), glycols (*p* = 0.92), sugars (*p* = 0.92), steroids (*p* = 0.059), phenols (*p* = 0.055), nucleotides (*p* = 0.053) and terpenes (*p* = 0.83; Figure 4).

### Effect of Mulching on the Microbial Community of *Camellia oleifera* Soil

In total, 479,866 pairs of reads in the amplified V3–V4 region 16S rRNA were identified by Illumina HiSeq analysis in all samples. A total of 901,051 sequences (valid tags) were processed, accounting for 94.7% of quantitative sequences. The mass readings in the soil samples ranged from 50,196 to 52,014 (Supplementary Table 2). These taxonomic sequences were then used to cluster operational taxons (OTUs) at a 3% similarity level and annotate OTUs using the Silva (bacterial) taxonomy database. A total of 1,106 OTUs were detected, and taxonomic analysis of species was performed (Supplementary Table 3). A Venn diagram shows that there were 897 OTUs in both the treatment and control groups. There was no significant difference in the number of unique OTUs between the control group and the treatment group (Supplementary Figure 2). The sampling work in the sampling analysis tends to reach the saturation platform (Supplementary Figure 3). With 97% sequence similarity, the hierarchical abundance curve method effectively covers the entire range of almost all bacterial diversity (Supplementary Figure 4).

**Figure 5:**
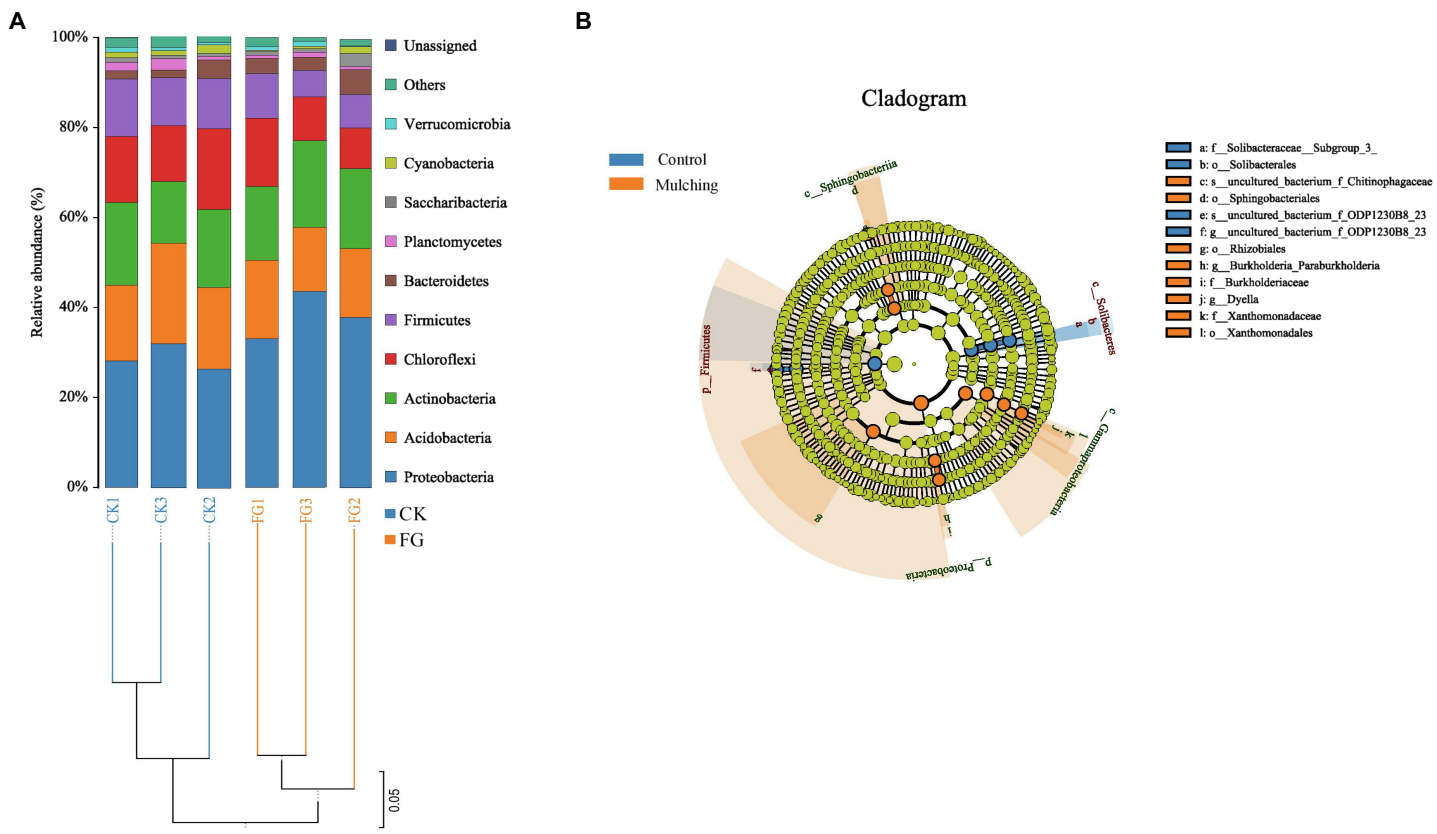
**(A)** Soil microbial community composition and sample cluster analysis in the ripening stage of *C. oleifera* after mulching; **(B)** linear discriminant analysis (LDA) effect size analysis of the evolutionary branch diagram. From the inside to the outside in the figure was the classification of phylum to species. Among them, the diameter of different dots was significantly positively correlated with species abundance. Yellow means that the species was not significant, while the significant species in the treatment group and the control group are represented by orange and blue, respectively.

The changes in the composition and diversity of the bacterial community in response to mulching were as follows:

All samples were divided into two categories in total (Figure 5A). The systematic analysis of the communities showed that only three systems (Proteobacteria, Acidobacteria, and Actinobacteria) were dominant (>60%) in all samples. The dominant bacteria at the phyum level (i.e., relative abundance) were mainly Proteobacteria, Acidobacteria, Actinobacteria, Chloroflexi, Firmicutes, Bacteroidetes, and Planctomycetes. Interestingly, the relative abundance of the Proteobacteria was higher than that of the control after mulching, while the relative abundances of the other two bacterial phyla were almost identical. Among the remaining bacterial phyla, the average relative abundance of Firmicutes and Chloroflexi was also lower under the mulched treatment than under the control. The number of OTUs of both groups ranged from 781 to 873, and the Shannon diversity index ranged from 5.2485 to 5.5184 (Supplementary Table 4). On average, the OTU (*p* > 0.74) and Shannon index (*p* < 0.37) were higher in the control soils than in the mulched group, and the remaining α-diversity indices were also higher in all the control groups but not significant.

Comparative assessment of microbial markers:

Significant differences in the abundance of bacterial biomarkers within each group were defined as a linear discriminant analysis (LDA) > 4 and value of *p* less than 0.05 (Supplementary Figure 5). The results revealed that 17 biomarkers were identified from all soil samples (Figure 5B). Among all taxonomic control-mulching levels, 6 biomarkers (p_Firmicutes, g_uncultured_bacterium_f_ODP1230B_23,o_Solibacterales,s_uncultured_bacterium_f_ODP12 30b8_23, c_Solibacteres and f_Solibacteraceae_Subgroup_3_) were associated with the CK (control) group and 11 biomarkers (p_Proteobacteria, o_Xanthomonadales, c_Gammaproteobacteria, o_Rhizobiales, f_ Xanthomonadaceae, g_Dyella, g_Burkholderia_Paraurkholderia, c_Sphingobacteriia, f_Burkholderiaceae, s_uncultured_bacterium_f_Chitinophagaceae, and o_Sphingo bacteriales) with the mulched group (Figure 5B).

### Correlation Analysis Between Soil Conditions and Plant Metabolism

**Figure 6:**
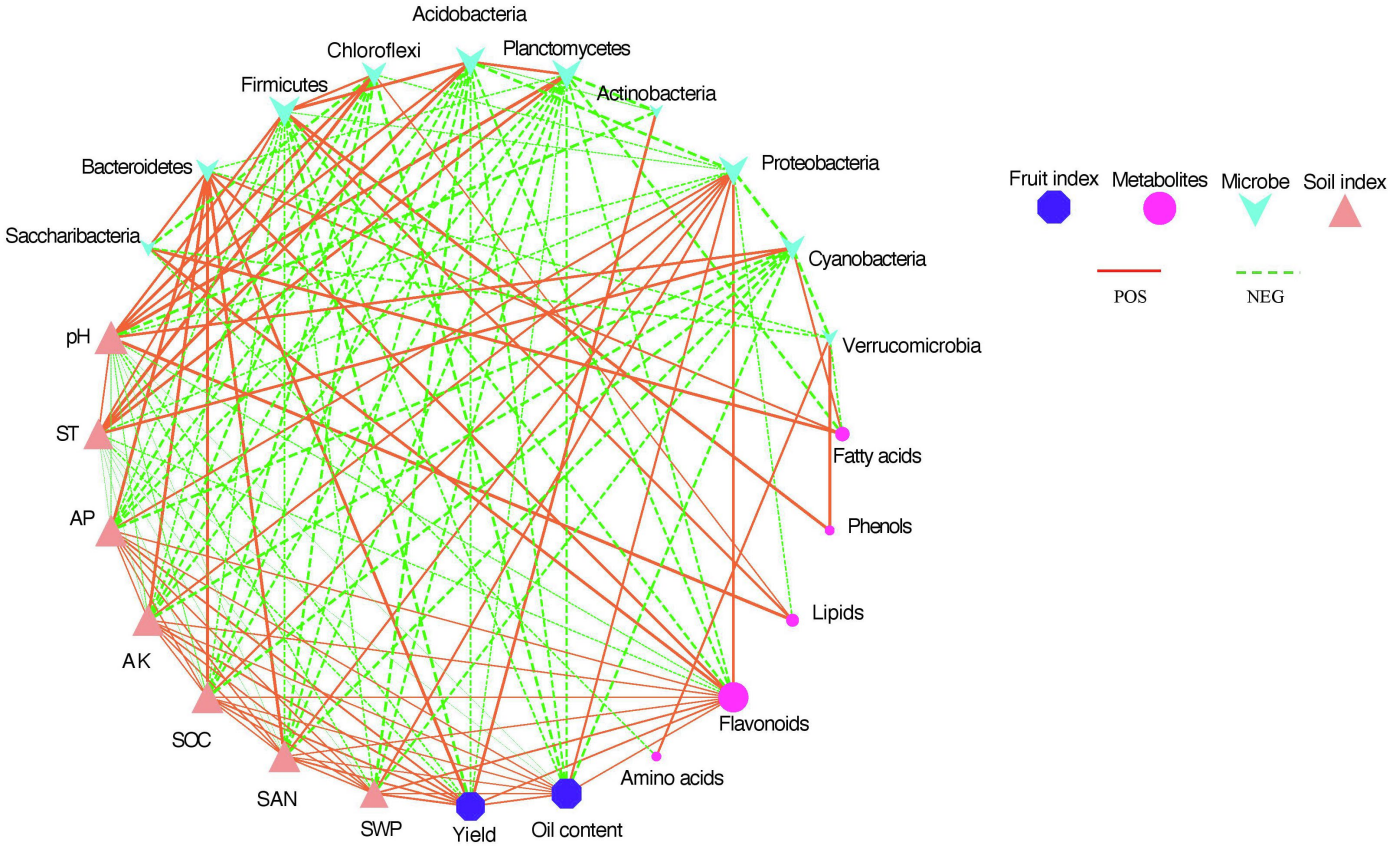
This analysis shows associations between soil microbial composition at the phylum level, fruit seed kernel metabolome (summed abundance of metabolite categories), soil nutrient indicators, and fruit yield indicators.

Figure 6 shows that the relationship of different indicators. The different shapes represent different indicators (AP, AK, SOC, and SAN, microorganisms, fruit yield and oil content, and seed kernel metabolites). The size of the shape represented the number of lines between nodes. The size of the shape represents how many lines are linked between different nodes. The larger the shape, the more indicator is correlated with other indicators. Green lines represented negative correlations, and red lines represented positive correlations. The thickness of the line was scaled to the degree of correlation, with thicker lines representing higher correlations. The bacterial phyla Proteobacteria, Bacteroidetes, and seed kernel metabolite flavonoids had the highest correlation with the other indicators. They were significantly and positively correlated with the soil nutrient indicators AP, AK, SOC, and SAN. In addition, soil nutrient indicators were negatively and significantly correlated with most of the soil microbial clades. Fruit oil content was positively and significantly correlated with Proteobacteria and soil nutrient indicators. The yield had a highly significant and positive correlation with Actinobacteria and Bacteroidetes. The results of the correlation plots showed that fruit yield traits were negatively and significantly correlated with most microorganisms. Soil temperature and pH were significantly and positively correlated with the microorganisms Firmicutes, Chloroflexi, Acidobacteria, Planctomycetes, and Cyanobacteria. However, soil temperature and pH were negatively correlated with soil nutrient indicators (AP, AK, SOC, and SAN). Flavonoids were the metabolites most correlated with all factors. Flavonoids were positively and significantly correlated with Proteobacteria, Bacteroidetes, Saccharibacteria, and soil nutrient indicators, except for all other soil microbial phyla that were negatively and significantly correlated.

## Discussion

The seed kernels of *C. oleifera* metabolites were identified from four stages of development by using untargeted GC–MS. We detected the metabolites including amino acids, organic acids, sugars, sugar alcohols, amines, carbohydrates, phenolics, nucleotides, terpenoids, flavonoids, and steroids. To identify differences in metabolite abundances at the four stages of *C. oleifera* fruit development, the multivariate statistical analysis method PLS-DA was allowed to analyze. The time periods S2–S3 (August–September) and S3–S4 (September–October) had the most differentially abundant metabolites, indicating that August to October was a period of critical metabolic changes for *C. oleifera* fruit development. Previous studies found that *C. oleifera* fruit development followed the S-type (Liang et al., 2019). July–August was the fruit expansion period when fruit volume increases, but the pericarp’s growth and development were the main focus of this stage. August–October was the oil conversion period, when crude fat content gradually increases with the maturation of seeds. In the late fruit ripening period, photosynthesis soluble sugars were converted into lipids. Only noradrenaline, aspartic acid, and phosphate were downregulated between fruit development in the 18 significantly different metabolites annotated, while the remaining 15 compounds were upregulated. Eighteen compounds were implicated in changes in 27 different metabolic pathways (Figure 2), with 11 pathways being most relevant. Most of these 11 pathways involved amino acid metabolism and carbon assimilation related to photosynthetic organisms, which may be due to seasonal drought stress. Seasonal drought stress changes several biochemical pathways related to amino acid biosynthesis or degradation.

We observed that metabolites could be clearly distinguished from the control after mulching during ripening of *C. oleifera* fruits (Supplementary Figure 1). The abundance of some amino acids (trans-ferulic acid, adenosine, 3-phenyllactic acid, tyrosine, tryptophan, abscisic acid, lipoic acid, etc.) was higher in the control group than in the mulched group according to our previous results (Ye et al., 2021a). The high levels of many amino acids were consistent over time with seasonal drought in the *C. oleifera* growing region. High amino acid levels were thought to enhance the plant response to adversity by affecting various physiological mechanisms, such as the regulation of osmotic changes, ROS detoxification, and the regulation of intracellular pH (Krasensky and Jonak, 2012). Studies have shown that in *Brassica napus*, free amino acids were increased under drought conditions (Good and Zaplachinski, 1994). However, the sum of all amino acids revealed no significant difference in abundance between the mulched and control groups (Figure 4). The same was true for the sum of sugars, steroids, carbohydrates, amines, phenols, and nucleotides. Alternatively, lipids, flavonoids, and organic acids were significantly different between the mulched and control groups. Plants respond to stress by progressively regulating their metabolism through continuous, transient, early, and late responses to metabolic changes. For example, high levels of cottonseed sugars and proline accumulate during days of salt exposure, drought, or cold (Krasensky and Jonak, 2012). The level of fatty acid and lipid accumulation in the metabolomic analysis was consistent with the measured fruit lipid content, with higher levels of lipids in the mulched group than in the control group. In contrast, organic acids and flavonoids were higher in the control group than in the mulched group. Studies have shown that organic acids and TCA cycle intermediates in glycogen increased under drought stress (Usadel et al., 2008). Flavonoids are specialized/secondary metabolites in plants and are used to defend against environmental stresses (e.g., biotic and abiotic stresses). Studies have shown that a high accumulation of flavonoids can improve drought tolerance in Arabidopsis (Nakabayashi et al., 2014). Together, the significantly higher accumulation of TCA cycle intermediates and flavonoids in the control group suggests that the control group trees has a more pronounced drought stress response than the mulched trees.

In nature, a variety of soil microorganisms was exposed to plants. One objective of this study was to investigate how plants respond physiologically and biochemically to these microbial supporters. The community composition and diversity of soil microbes are widely applied as bioindicators of soil qualities and bacteria are the most diverse groups of soil microorganisms (Farmer et al., 2017). Plant adaptation in drought and nondrought environments is closely linked to soil bacterial composition and to water availability (Lau and Lennon, 2012). Yang (2019) showed that soil bacteria in southern (China) red soil hilly areas were significantly influenced by soil moisture status. The water deficiency limited microbial growth and organic matter decomposition, which then caused negative feedback to the woodland ecosystem, and nitrogen cycle (Fierer and Schimel, 2003). Previous studies have shown that mulching and fertilizing alter the soil bacterial community (Tiquia et al., 2002). In this study, mulching caused changes in soil bacterial communities (Figure 5A) and improved plant performance (with growth and fruit yield; Figure 3), and these were mainly attributed to the physicochemical properties of soil induced by treatment. This study found that mulching reduced the diversity of soil bacteria, improved soil biology and soil nutrition, in accordance with previous studies (Fu et al., 2019). LEfSe analysis found discriminatory biomarkers between the rhizosphere soil of the control and mulched groups (Figure 5B). In the mulched treatment soils, more biomarkers were found than in the control group. The most functional and active soil microorganisms in our cropping system were Proteobacteria, Actinobacteria, Bacteroidetes and Acidobacteria, Chloroflexi, and Firmicutes based on the differences between the genetic indications in the various cropping systems. This was in line with the findings of Song (Li and Wu, 2018) who discovered similar bacterial species under a variety of plant species, implying that these microbial communities play an important role in the soil microbiota. For example, Proteobacteria in the dominant bacteria is highly responsive to N in the soil. This was also verified by the positive correlation between Proteobacteria and available nitrogen in our co-expression network. Proteobacteria is the largest phylum of bacteria, many of which can perform nitrogen fixation and adapt to various complex environments (Liu et al., 2014; Luo, 2014). Nitrogen is a key factor affecting soil ecosystems and biogeochemical cycles. Fierer et al. (2012) suggested that nitrogen levels may directly or indirectly induce shifts in major microbial community members, especially Proteobacteria and Bacteroidetes.

Fruit yield at maturity was significantly higher for mulched than control for two consecutive years, and fruit yield in the second year was higher than the first year. It is plausible that fruit yield will continue to increase with increasing mulching time. Arabidopsis plants treated with soil microbes showed an increase in biomass (Badri et al., 2013), suggesting that soil microbes (regardless of their composition and identity) are favorable for plant growth. Furthermore, previous studies found that an increase in soil abundant microorganisms or certain microbial species’ diversity can positively affect plant biomass (Maherali and Klironomos, 2007). These studies partially explain our observation of why mulching increases in the abundance of certain microorganisms in the soil, promoting plant growth. Although the soil water potential of the mulching treatment at the ripening stage was greater than that of the control, the difference between the two was not significant. This is because the period of *C. oleifera* fruit ripening was the rainy season in the planting area. However, the ecological mats had the effect of preventing water evaporation due to their material characteristics, so the mulched group’s water potential was slightly higher than that of the control group but not significant. Soil temperature and soil nutrient indicators (SAN, AP, AK, and SOC) in the mulched treatment group were significantly higher than those in the control group, possibly due to the crops dropped down from the tree used to gradually degrade the ecological mats and increase the nutrients in the soil. Ecological mats are made from a variety of plant materials and are organic mulches along with straw. They have similar principles of action on the soil after mulching. Straw mulching increased the number of soil microorganisms because straw mulching alleviated drastic fluctuations in soil moisture and temperature (Zhang et al., 2015). Straw mulching also increased the type and number of root secretions, which gave soil microorganisms more carbon and energy sources and increased root growth in the crop (Zhang et al., 2015).

Strong relationships existed between soil microbial community, metabolites, soil properties, and tree phenotype measures (Figure 6). Although it was not possible to determine causality from these relationships, this analysis provides some basis for future research. For example, it was observed that *C. oleifera* fruit yield and oil content were positively correlated with several dominant bacterial phyla including Aspergillus, Proteus, and Firmicutes (Figure 6). Aspergillus can contribute to plant growth through nutrient acquisition and increased disease protection. By improving the acquisition and protection of disease, Proteus can support the growth of plants. Second, Proteus was involved in producing two major greenhouse gases, methane, and nitrous oxide. γ- and β-Proteus (Pseudomonas, Burkholderia, Xanthomonas) and Firmicutes (lactobacilli) were considered the most active groups with disease inhibition (Mendes et al., 2011). This could be one of the possible reasons for the improved plant yield after mulching. It has also been shown that plant growth-promoting bacteria can enhance host tolerance by increasing gene expression associated with drought tolerance. The soil pH was likely closely related to microbial composition and soil properties. The *C. oleifera* growing area is an acidic red soil, but the soil pH after mulching was lower than that of the control group. A similar study showed that organic mulching reduced soil pH in *Emblica officinalis* Gaertn (Kumar, 2014). The decrease in soil pH may be due to the increase in organic matter from decomposing mulch or microbial biomass releasing organic content. This study revealed that soil pH was a main factor significantly associated with several key phyla (Planctomycetes, Firmicutes, Chloroflexi, Acidobacteria, Cyanobacteria), and lipid metabolites (Figure 6), followed by soil temperature. Previous reports have shown that a number of bacterial events, such as ammonia oxidation and phosphate solubilization, depend on pH (Mohabeer et al., 1997), and the phylum Acidobacteria contains many pH-dependent taxa (Zhou et al., 2017; Song et al., 2018). Together these results show the important role that soil pH plays in microbial activity and plant growth, and whether it causes improved plant growth, or a result of other factors is unclear from this study. In conclusion, positive correlations between fruit yield, certain soil microbiota, and several soil physical properties were found. Similarly, there was a strong positive correlation between metabolites (flavonoids, fatty acids, lipids) in seed kernels and certain dominant bacteria. This study supports the interaction of soil microbial communities in plant growth and is important to consider attempts to improve agricultural yield. It is worth mentioning that the observed answers could be associated with further unknown soil factors or interactions. It is also unclear how the soil microbial community causes the metabolites of the fruit seed kernel to change. This role may involve a combination of microbial signals (bioactive molecules) and transduction pathways of a plant signal.

This study showed that organic mulching increased fruit and oil yields in *C. oleifera* compared to unmulched trees. GC-TOF analysis of seed kernels revealed that under the condition of seasonal drought, the most significant difference in seed kernel metabolism occurred in the period of high-speed oil conversion (August–October). Organic mulching effects on soil diversity and the community structure around *C. oleifera* have revealed that organic mulching has changed the relative abundance of dominant bacteria in soil. Soil physical properties were also significantly different under mulched conditions. Soil pH and temperature were the two indicators with the most correlations with soil microbiota. The flavonoids of seed metabolites were the most correlated with soil conditions (nutrient conditions, microbiota, and biochemical conditions). The yield of *C. oleifera* (oil content and yield per crown area) was significantly positively correlated with soil microbial Proteobacteria, Bacteroidetes, and soil nutrition indexes. Therefore, organic mulching leads to a better microecological environment conducive to better growth and overall yield of *C. oleifera*. Combined with the improvement of *C. oleifera* yield, organic mulching was an effective measure to improve protection from seasonal drought.

## Data Availability Statement

The data presented in the study are deposited in the NCBI repository, accession number PRJNA698393 (Link: https://www.ncbi.nlm.nih.gov/).

## Author Contributions

HY: conceptualization, investigation, methodology, formal analysis, visualization, and writing—original draft. MG: metabolic data analysis and language editing. YW, ZC, TZ, SL, and YZ: reviewing and editing. SS: conceptualization, funding acquisition, and supervision. All authors contributed to the article and approved the submitted version.

## Funding

This work was supported by National Key Research and Development Program (Grant no. 2019YFD1002401).

## Conflict of Interest

The authors declare that the research was conducted in the absence of any commercial or financial relationships that could be construed as a potential conflict of interest.

## Publisher’s Note

All claims expressed in this article are solely those of the authors and do not necessarily represent those of their affiliated organizations, or those of the publisher, the editors and the reviewers. Any product that may be evaluated in this article, or claim that may be made by its manufacturer, is not guaranteed or endorsed by the publisher.

## Footnotes

^1^ http://huttenhower.sph.harvard.edu/galaxy/

^2^ https://www.omicsolution.org/wkomics/main/

## Supplementary Material

Supplementary Figure 1 S1–S2, S2–S3, and S3–S4 pairwise comparison of 2D scores loading plot.

Supplementary Figure 2 Venn diagram the number of common and unique OTUs between the coverage and control groups.

Supplementary Figure 3 Shannon index curves.

Supplementary Figure 4 Rank abundance curve.

Supplementary Figure 5 Linear discriminant analysis.

Supplementary Table 1 S1-S2, S2-S3, S3-S4 compare the name of the pathway involved in the important metabolites screened out, the total number of metabolites, hits, the Raw *P* value, log10(*P*) and FDR.

Supplementary Table 2 Description of all identified sequences affiliated with soil samples using 16S rRNA Illumina HiSeq analysis after experiments in 2017.

Supplementary Table 3 Species taxonomy analysis affiliated with soil samples using Illumina HiSeq analysis.

Supplementary Table 4 Alpha diversity of soil microbial communities in *C. oleifera* at ripening after mulching.
