# Supplementary material for: Relationship of Soil Microbiota to Seed Kernel Metabolism in Camellia oleifera Under Mulched
Source: Front Plant Sci. 2022 Jun 20;13:920604. doi: 10.3389/fpls.2022.920604 (PMC9251579; doi:10.3389/fpls.2022.920604)
Supplement: Supplementary Figure 1 — S1–S2, S2–S3, and S3–S4 pairwise comparison of 2D scores loading plot. [file Table_1.docx]

Supplementary Material

**Relationship of Soil Microbiota to Seed Kernel Metabolism in *Camellia oleifera* under Mulched**

**Honglian Ye^1, 2,^ Yue Wen^3^, Zhigang Chen^4^, Taikui Zhang^5^, Shengxing Li^6^, Menglong Guan^7^, Yunqi Zhang^8^, Shuchai Su^1^***

^1^ Key Laboratory for Silviculture and Conservation, Ministry of Education, Beijing Forestry University, Beijing 100083, P.R.China；

^2^ West Coast Metabolomics Center, UC-Davis, 95616 CA, USA

^3^ Research Center for Xinjiang Characteristic Fruit Tree, College of Forestry & Horticulture, Xinjiang Agricultural University, 830052, Urumqi, P.R.China；

^4^ State Key Joint Laboratory of Environmental Simulation and Pollution Control, School of Environment, Tsinghua University, Beijing 100084, P.R.China

^5^ Ministry of Education Key Laboratory of Biodiversity Science and Ecological Engineering, School of Life Sciences, Fudan University, Shanghai, China

^6^ Camphor Engineering Technology Research Center for State Forestry Administration, Jiangxi Academy of Forestry, Nanchang 330032, P.R.China；

^7^ West China Hospital of Sichuan University, Chengdu, 610041 P.R.China；

^8^ Beijing Academy of Forestry and Pomology Sciences, Beijing 100083, P.R.China；


Supplementary Figure 1. S1-S2, S2-S3 and S3-S4 pairwise comparison of 2D scores loading plot.

 Supplementary Figure 2. Venn diagram the number of common and unique OTUs between the coverage and control groups.

Supplementary Figure 3. Shannon index curves

Supplementary Figure 4. Rank abundance curve

Supplementary Figure 5. Linear Discriminant Analysis

Supplementary Table1. S1-S2, S2-S3, S3-S4 compare the name of the pathway involved in the important metabolites screened out, the total number of metabolites, hits, the Raw *P* value, log10(*P*) and FDR

| Pathway name | Total | Hits | Raw *P* | LOG10 (*P*) | FDR |
| --- | --- | --- | --- | --- | --- |
| Carbon fixation in photosynthetic organisms | 21 | 3 | 3.76E-04 | 3.42 | 2.08E-02 |
| Alanine, aspartate and glutamate metabolism | 22 | 3 | 4.34E-04 | 3.36 | 2.08E-02 |
| Glycine, serine and threonine metabolism | 33 | 3 | 1.47E-03 | 2.83 | 3.52E-02 |
| Citrate cycle (TCA cycle) | 20 | 2 | 8.84E-03 | 2.05 | 1.41E-01 |
| Pyruvate metabolism | 22 | 2 | 1.07E-02 | 1.97 | 1.46E-01 |
| Glycolysis / Gluconeogenesis | 26 | 2 | 1.48E-02 | 1.83 | 1.77E-01 |
| Glyoxylate and dicarboxylate metabolism | 29 | 2 | 1.82E-02 | 1.74 | 1.94E-01 |
| Cysteine and methionine metabolism | 46 | 2 | 4.33E-02 | 1.36 | 3.82E-01 |
| C5-Branched dibasic acid metabolism | 6 | 1 | 4.38E-02 | 1.36 | 3.82E-01 |
| Glycerolipid metabolism | 21 | 1 | 1.37E-03 | 2.86 | 3.52E-02 |
| Lysine biosynthesis | 9 | 1 | 7.18E-03 | 2.14 | 1.38E-01 |
| Ascorbate and aldarate metabolism | 18 | 2 | 6.50E-02 | 1.19 | 5.20E-01 |
| Nicotinate and nicotinamide metabolism | 13 | 1 | 9.26E-02 | 1.03 | 6.84E-01 |
| Tyrosine metabolism | 16 | 1 | 1.13E-01 | 0.95 | 7.12E-01 |
| Butanoate metabolism | 17 | 1 | 1.19E-01 | 0.92 | 7.12E-01 |
| Arginine biosynthesis | 18 | 1 | 1.26E-01 | 0.90 | 7.12E-01 |
| beta-Alanine metabolism | 18 | 1 | 1.26E-01 | 0.90 | 7.12E-01 |
| Monobactam biosynthesis | 8 | 2 | 1.46E-01 | 0.84 | 7.24E-01 |
| Thiamine metabolism | 22 | 1 | 1.52E-01 | 0.82 | 7.24E-01 |
| Valine, leucine and isoleucine biosynthesis | 22 | 1 | 1.52E-01 | 0.82 | 7.24E-01 |
| Pantothenate and CoA biosynthesis | 23 | 1 | 1.58E-01 | 0.80 | 7.24E-01 |
| Phosphatidylinositol signaling system | 26 | 1 | 1.77E-01 | 0.75 | 7.46E-01 |
| Galactose metabolism | 27 | 1 | 1.84E-01 | 0.74 | 7.46E-01 |
| Inositol phosphate metabolism | 28 | 1 | 1.90E-01 | 0.72 | 7.46E-01 |
| Cyanoamino acid metabolism | 29 | 1 | 1.96E-01 | 0.71 | 7.46E-01 |
| Terpenoid backbone biosynthesis | 30 | 1 | 2.02E-01 | 0.69 | 7.46E-01 |
| Aminoacyl-tRNA biosynthesis | 46 | 1 | 2.94E-01 | 0.53 | 1.00E+00 |

Supplementary Table 2. Description of all identified sequences affiliated with soil samples using 16S rRNA Illumina HiSeq analysis after experiments in 2017.

| Sample ID | PE Reads | Raw Tags | Clean Tags | Effective Tags | Avg Len (bp) | GC (%) | Q20 (%) | Q30 (%) | Effective (%) |
| --- | --- | --- | --- | --- | --- | --- | --- | --- | --- |
| CK1 | 79,801 | 67,159 | 52,603 | 52,014 | 410 | 56.81 | 95.49 | 91.13 | 65.18 |
| CK2 | 79,790 | 65,664 | 51,257 | 50,789 | 412 | 56.21 | 95.36 | 90.98 | 63.65 |
| CK3 | 79,923 | 66,741 | 52,143 | 51,536 | 410 | 56.62 | 95.48 | 91.15 | 64.48 |
| FG1 | 80,035 | 68,196 | 53,321 | 50,939 | 411 | 56.67 | 95.41 | 91.03 | 63.65 |
| FG2 | 79,926 | 66,706 | 52,210 | 51,402 | 412 | 55.6 | 95.42 | 91.14 | 64.31 |
| FG3 | 80,391 | 69,301 | 54,505 | 50,196 | 413 | 56.26 | 95.41 | 91.08 | 62.44 |

Supplementary Table 3. Species taxonomy analysis affiliated with soil samples using Illumina HiSeq analysis.

| Sample | Kingdom | Phylum | Class | Order | Family | Genus | Species |
| --- | --- | --- | --- | --- | --- | --- | --- |
| CK1 | 44,288 | 44,288 | 44,274 | 43,722 | 42,984 | 39,121 | 28,291 |
| CK2 | 42,573 | 42,573 | 42,521 | 41,920 | 39,491 | 37,111 | 28,652 |
| CK3 | 43,705 | 43,705 | 43,682 | 43,306 | 42,579 | 38,162 | 27,333 |
| FG1 | 38,576 | 38,576 | 38,569 | 38,332 | 37,814 | 35,590 | 24,710 |
| FG2 | 44,454 | 44,454 | 44,370 | 43,716 | 42,689 | 39,600 | 22,596 |
| FG3 | 37,121 | 37,121 | 37,100 | 36,760 | 36,322 | 33,166 | 17,925 |

Note: The classification of microbial species is generally divided into seven levels: Domain, phylum, class, order, family, genus, and species, and each OTU represents a set of classification levels for a certain type.

| Sample ID | OTU | Chao1 | Simpson | Shannon | Coverage |
| --- | --- | --- | --- | --- | --- |
| Control_1 | 873 | 917.2642 | 0.0118 | 5.4109 | 0.9984 |
| Control_2 | 820 | 842.9412 | 0.0102 | 5.5184 | 0.9991 |
| Control_3 | 820 | 881.62 | 0.0143 | 5.2849 | 0.9982 |
| Mulching_1 | 807 | 862.1549 | 0.0104 | 5.4081 | 0.9977 |
| Mulching_2 | 781 | 809.5938 | 0.0112 | 5.2485 | 0.9986 |
| Mulching_3 | 839 | 917.0455 | 0.0107 | 5.4209 | 0.9973 |

Supplementary table 4. Alpha diversity of soil microbial communities in C. oleifera at ripening after mulching
